# Cardiomyopathy as presenting sign of glycogenin-1 deficiency—report of three cases and review of the literature

**DOI:** 10.1007/s10545-016-9978-1

**Published:** 2016-10-07

**Authors:** Carola Hedberg-Oldfors, Emma Glamuzina, Peter Ruygrok, Lisa J. Anderson, Perry Elliott, Oliver Watkinson, Chris Occleshaw, Malcolm Abernathy, Clinton Turner, Nicola Kingston, Elaine Murphy, Anders Oldfors

**Affiliations:** 1 Department of Pathology and Genetics, University of Gothenburg, Gothenburg, Sweden; 2 National Metabolic Service, Starship Children’s Hospital, Auckland, New Zealand; 3 Green Lane Cardiovascular Service, Auckland City Hospital, Auckland, New Zealand; 4 Department of Cardiology, St George’s Hospital, London, UK; 5 Barts Heart Centre, London, UK; 6 Wakefield Heart Centre, Wakefield Hospital, Wellington, New Zealand; 7 Anatomical Pathology, LabPlus, Auckland City Hospital, Auckland, New Zealand; 8 Charles Dent Metabolic Unit, National Hospital for Neurology and Neurosurgery, London, UK

## Abstract

We describe a new type of cardiomyopathy caused by a mutation in the glycogenin-1 gene (*GYG1*). Three unrelated male patients aged 34 to 52 years with cardiomyopathy and abnormal glycogen storage on endomyocardial biopsy were homozygous for the missense mutation p.Asp102His in *GYG1*. The mutated glycogenin-1 protein was expressed in cardiac tissue but had lost its ability to autoglucosylate as demonstrated by an in vitro assay and western blot analysis. It was therefore unable to form the primer for normal glycogen synthesis. Two of the patients showed similar patterns of heart dilatation, reduced ejection fraction and extensive late gadolinium enhancement on cardiac magnetic resonance imaging. These two patients were severely affected, necessitating cardiac transplantation. The cardiomyocyte storage material was characterized by large inclusions of periodic acid and Schiff positive material that was partly resistant to alpha-amylase treatment consistent with polyglucosan. The storage material had, unlike normal glycogen, a partly fibrillar structure by electron microscopy. None of the patients showed signs or symptoms of muscle weakness but a skeletal muscle biopsy in one case revealed muscle fibres with abnormal glycogen storage. Glycogenin-1 deficiency is known as a rare cause of skeletal muscle glycogen storage disease, usually without cardiomyopathy. We demonstrate that it may also be the cause of severe cardiomyopathy and cardiac failure without skeletal muscle weakness. *GYG1* should be included in cardiomyopathy gene panels.

**Electronic supplementary material:**

The online version of this article (doi:10.1007/s10545-016-9978-1) contains supplementary material, which is available to authorized users.

## Introduction

Glycogen storage diseases (GSD) are important causes of myopathy and cardiomyopathy and are in most instances the consequence of a deficiency of an enzyme involved in glycogen metabolism. There are two types of muscle glycogenoses, those with defective degradation of normal glycogen (such as GSD II, III, V, VII, IX) and those with defective synthesis of glycogen (GSD 0, IV, XV) (DiMauro and Spiegel 2011; Oldfors and DiMauro 2013; Hedberg-Oldfors and Oldfors 2015).

Muscle GSD associated with deficiency of enzymes involved in glycogen synthesis include glycogen synthase (*GYS1*; GSD 0), branching enzyme (*GBE1*; GSD IV) and glycogenin-1 (*GYG1*; GSD XV). Glycogenin-1 is an autoglucosylating enzyme serving as a primer for glycogen synthase and branching enzyme by the formation of an oligosaccharide consisting of 8–14 glucose molecules, which is bound to glycogenin-1 by a glucose-O-tyrosyl linkage at position 195 (Lomako et al 2004; Roach et al 2012). Previously described individuals with GSD XV had pure myopathy except one patient who also had cardiac involvement with arrhythmias (Moslemi et al 2010; Malfatti et al 2014; Colombo et al 2016; Fanin et al 2015; Luo et al 2015; Akman et al 2016).

We describe three patients with cardiomyopathy and heart failure necessitating cardiac transplantation in two. Investigations revealed massive storage of abnormal glycogen in the heart and a homozygous missense mutation in *GYG1* resulting in a nonfunctional glycogenin-1 in all patients.

## Patients and methods

A summary of the results from clinical, radiological and laboratory examinations are presented in Table 1. The study complies with the Declaration of Helsinki and informed consent has been obtained from the patients.

**Table 1:** Results of clinical, radiological and laboratory examinations

	Patient 1	Patient 2	Patient 3
Gender	Male	Male	Male
Descent	New Zealand/British	British	British
Age, years	49	52	34
Age of onset, years	34	46/50	23
Initial symptoms	Shortness of breath, chest pain on exertion, lethargy and palpitations	CVA/Chest pain, sweatiness and palpitations	Shortness of breath and chest pain
ICD	Age 45 years	Age 49 years	Age 31 years
Cardiac MRI (with gadolinium)	Severely impaired left ventricular function with severe dilatation. Thinned out myocardium in the posterolateral wall showing almost full thickness scarring. Diffusely patchy scarring mostly in the epicardial and mid-myocardial regions.	Severely dilated LV with a large area of thinning and akinesis affecting the entire lateral wall from base to apex (anterolateral, anterior and inferolateral walls). Other regions were hypertrophied with preserved systolic function. Extensive late gadolinium enhancement in the entire thinned region.	Very extensive (>50 %) late gadolinium enhancement in a non-ischaemic pattern. Moderate LV impairment. Maximum LV wall thickness of 23 mm.
ECG (before ICD insertion)	Sinus rhythm. Widespread T wave inversion. QRS duration 120 ms	Sinus rhythm with poor lateral R wave progression. QRS duration 126 ms. QTc normal.	T wave inversion in leads I, II, III, AVF and V6 with ST elevation in leads V3-V6. QRS duration 102 ms. QTc normal.
Echocardiogram	Age 48 years	Age 50 years	Age 32 years
Left ventricular end-diastolic volume (ml)	281	277	151
Septal wall thickness (cm)	1,4	1,6	1,6
Posterior wall thickness (cm)	0,9	1,0	1,4
Ejection fraction (%)	25–30	30–35	59
Coronary angiogram	Normal	Normal	Normal
Heart transplantation	Age 48 years	Age 52 years	No
Endomyocardial biopsy Light microscopy	PAS-positive inclusions partial removal by diastase	PAS-positive inclusions partial removal by diastase	PAS-positive inclusions
Electron microscopy	Normal glycogen and filamentous or amorphous material	Not performed	Storage of non-lysosomal glycogen
Muscle biopsy	Variability of fibre size and scattered fibres with accumulation of PAS-positive inclusions	Not performed	Not performed
Skeletal muscle strength	Normal	Normal	Normal
GYG1 mutation	Homozygous c.304G > C, p.(Asp102His)	Homozygous c.304G > C, p.(Asp102His)	Homozygous c.304G > C, p.(Asp102His)

*ICD* Implantable cardioverter-defibrillator, *CVA* Cerebral vascular accident, *MRI* Magnetic resonance imaging, *LV* Left ventricle, *ECG* Electrocardiography, *PAS* Periodic acid-Schiff

### Patient 1

This 49-year-old man was of New Zealand-British descent. He presented to cardiac services at 36 years of age with palpitations and exertional chest pain. For approximately 2 years prior he had episodes of calf pain, with increasing shortness of breath on exertion and lethargy. He had previously been very active. He had normal intellect, no history of recorded hypoglycaemia or symptoms to suggest this, a normal birth and childhood and no other medical problems. His mother and father died in their 70s of a cerebrovascular accident and bowel cancer respectively. He has an older brother and sister and two sons, all of whom are well. His echocardiogram at presentation revealed severe cardiac hypertrophy with speckling of the cardiac muscle and an ejection fraction of 44 %. A cardiac muscle biopsy was performed and glycogen accumulation was seen (see below).

He had no clinical evidence of skeletal muscle disease or weakness, but a mildly elevated serum creatine kinase (s-CK) on one occasion. Muscle biopsy showed an abnormal glycogen pattern (see below) and a liver biopsy showed microvesicular steatosis and glycogen accumulation with mild inflammation. He had no hepatomegaly and his liver enzyme tests and liver ultrasound scans were normal.

**Fig. 1:**
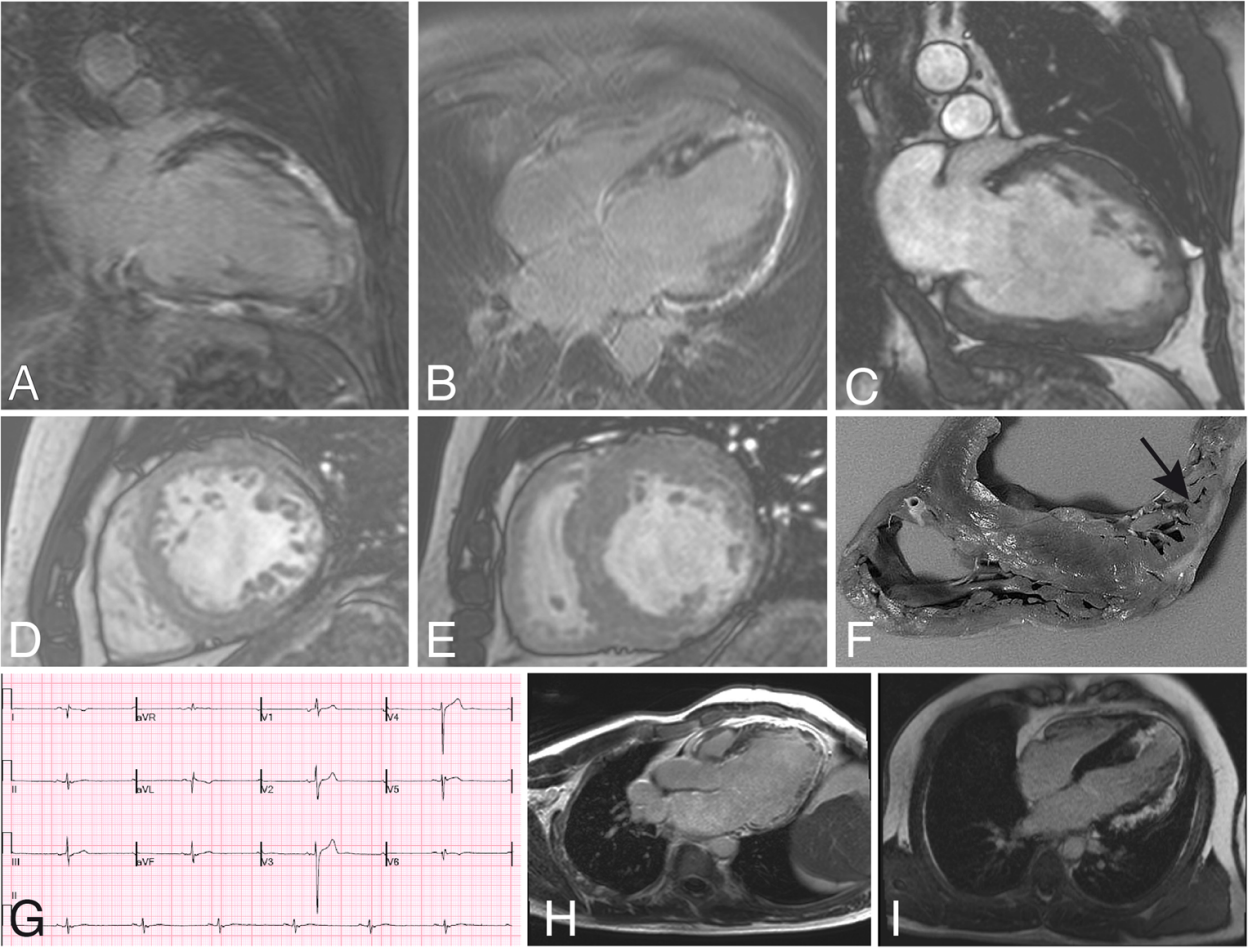
Cardiac MRI (A-E) and explant (F) of patient 1, ECG (G) and cardiac MRI (H) of patient 2 and cardiac MRI (I) of patient 3. **a** A 2-chamber vertical long-axis delayed enhancement image of the LV demonstrating very extensive increased signal in the inferior surface, the distal half of the diagonal surface, and the LV apex, predominantly in the mid-myocardial and subepicardial layers of the myocardium. The abnormal signal indicates the presence of fibrosis or scar tissue. **b** A 4-chamber long-axis delayed enhancement image of the LV, demonstrating extensive abnormal enhancement consistent with fibrosis or scar formation in the mid-myocardial and subepicardial layers of the myocardium, involving the entire obtuse marginal surface, the apex and the posterior ventricular septum. **c** A 2-chamber vertical long-axis cine frame of the LV at end-diastole, which is dilated and globular in shape. There is variable wall thickness, with thinning of the distal half of the diagonal surface and the apex. An increased number of trabeculations in these regions indicates localized non-compaction of the myocardium. **d** A short-axis cine frame of the middle third of the LV at end-diastole shows thinning of the obtuse marginal surface, with markedly increased trabeculation of both this region, and, albeit to a lesser extent, of the diagonal and anterior septal myocardium also. **e** A short-axis cine frame of the basal LV at end-systole demonstrates variable increase in wall thickening in the ventricular septum and diagonal surfaces, with marked wall thinning in the posterior obtuse marginal and inferior surfaces. **f** Transverse section of the explanted heart including the right ventricle interventricular septum and parts or the left ventricle demonstrating variable thickening of the septum and thin and trabeculated posterior wall (arrow). **g** ECG of patient 2 demonstrating sinus rhythm with very poor R wave progression in the lateral leads. **h** Cardiac MRI of patient 2 demonstrating a markedly dilated left ventricle with thin lateral wall with late gadolinium contrast enhancement. **i** Cardiac MRI of patient 3 demonstrating extensive late gadolinium contrast enhancement

He progressed to develop increasing fatigue and lethargy, worsening cardiac hypertrophy with secondary dilatation and worsening ejection fraction (28 % at 44 years). At age 45 years a cardiac MRI (Fig. 1a-e) demonstrated a severely dilated left ventricle with moderate-severely impaired ventricular function. Right ventricular function was normal without any focal thickening or hypertrophy. Regional wall motion assessment showed a complex pattern of akinesis. There was delayed gadolinium enhancement showing an extensive pattern of scarring with a patchy appearance, mostly in the mid-myocardial or sub-epicardial segments. The myocardium was thinned out in the posterolateral wall with almost full thickness scarring. Coronary angiogram at 45 years was normal.

He developed increasing issues with cardiac arrhythmia and an ICD was implanted. He had many episodes of anti-tachycardia pacing and three shocks.

He was listed for cardiac transplantation at 48 years of age and underwent a cadaveric donor transplant 1 month later. He had no significant complications and no clinical evidence of multi-system disease at the last examination 6 months post transplantation.

### Patient 2

This 52-year-old man presented at age 46 years with left-sided weakness, 2 days after a boating accident. He had a background history of hypertension and dyslipidemia. A right middle cerebral artery infarct was diagnosed. He was treated with thrombolysis and made a good recovery. An echocardiogram at the time showed a thickened interventricular septum (1.3 cm), a mildly dilated left ventricle, and impaired systolic function with an estimated ejection fraction of 40–50 %.

He had a normal birth and childhood. There were no other specific medical issues. The family is non-consanguineous of English background. He has three siblings—an elder brother in his 50s with type 2 diabetes and two younger sisters, in their 40s. None of his siblings have known cardiac disease. A paternal grandfather died from a myocardial infarction aged 63 years. A paternal aunt had a valve replacement. There is no other cardiac history within the family.

He remained well until aged 50 years when he developed palpitations, sweatiness and chest pain whilst driving. He was found to have ventricular tachycardia with presyncope and ECG showed sinus rhythm with markedly poor lateral R wave progression (Fig. 1g). An echocardiogram showed severe left ventricular dilatation. Systolic function was severely impaired with an estimated ejection fraction of 30–35 %. There was akinesis of the inferolateral wall and apical lateral wall and hypokinesis of the anterolateral and anterior wall. There was severe left ventricular diastolic dysfunction and moderate left atrial dilatation with mild mitral regurgitation. Cardiac MRI (Fig. 1h) confirmed that the left ventricle was severely dilated with a large area of thinning and akinesis affecting the entire lateral wall from base to apex (anterolateral, anterior and inferolateral walls). Other regions were hypertrophied with preserved systolic function. Late enhancement following gadolinium contrast was seen in the entire thinned region.

On examination, his weight was 81.2 kg. He did not have palpable hepatomegaly. Skeletal muscle strength was normal. An EMG was also normal. Blood glucose and lactate levels were normal. Blood film showed no vacuolation or increase in lymphocyte glycogen content. Red blood cell glycogen level was also normal at 22ug/g Hb (RR: 10–120). An acylcarnitine profile was normal. Creatine kinase was 88 IU/L (RR: 38–204). No urinary glucose tetrasaccharide was detected.

The patient remained symptomatic with shortness of breath on exertion. An ICD was inserted aged 49 years and a left ventricular assist device aged 51 years. Orthotopic cardiac transplant was performed aged 52 years. Initial post-operative course was complicated by renal impairment requiring haemofiltration. He is now well 6 months post-transplant.

### Patient 3

This 34-year-old man of white UK background first presented aged 23 years with shortness of breath and chest pain. Prior to this he had been well, with active participation in cross-country running and competitive football. A diagnosis of hypertrophic cardiomyopathy was made. An ICD was fitted aged 31 years and current medications include bisoprolol, spironolactone and warfarin. He has never had skeletal muscle weakness or symptoms suggestive of hypoglycaemia.

He reported a normal birth and childhood. He has two siblings, a brother aged 39 years has been screened by echocardiography and has no cardiac disease, a sister aged 29 years has opted not to be screened. Both siblings are clinically well. A maternal uncle has hypertrophic cardiomyopathy of unknown cause (no endocardial biopsy or genetic studies performed).

On examination, he had no hepatomegaly and muscle strength was normal. ECG, echocardiogram and cardiac MRI findings were as in Table 1 and demonstrated hypertrophic cardiomyopathy with moderate to severe LVH, mild RVH and marked late gadolinium enhancement (Fig. 1i).

Blood glucose and lactate levels were normal. Creatine kinase was 189 IU/L (RR: 38–204). Red blood cell glycogen level was normal at 22 μg/g Hb (RR: 10–120). Excess urinary glucose tetrasaccharide was not detected.

### Morphological and immunohistochemical analysis

Endomyocardial biopsy from the right ventricle was performed at the age of 48 years (patient 1), 49 years (patient 2) and 33 years (patient 3). The specimens were fixed in paraformaldehyde for paraffin embedding or glutaraldehyde for electron microscopy. Open skeletal muscle biopsy of the quadriceps muscle was performed at the age of 37 years (patient 1). Specimens were snap frozen for cryostat sectioning and histochemistry. Standard techniques were applied for histochemical staining.

### Molecular genetic analysis

DNA from all three patients was analysed by sequencing a panel of genes associated with muscle glycogen storage diseases (*AGL*; *ALDOA*; *ENO3*; *FBP2*; *GAA*; *GBE1*; *GYG1*; *GYS1*; *LDHA*; *PFKM*; *PGAM2*; *PGK1*; *PHKA1*; *PHKG1*; *PYGM*) (Sheffield Diagnostics Genetics Service, UK). Patients 2 and 3 also had mutation analysis of the *LAMP2* and *PRKAG2* genes prior to the panel sequencing but no pathogenic variants were detected. Patient 3 in addition had a cardiomyopathy gene panel investigation including 40 genes but only a *MYH6* variant of uncertain importance was detected (Sheffield Diagnostics Genetics Service, UK). This variant was also detected in his asymptomatic mother.

### Whole-exome sequencing

Whole-exome sequencing (WES) was performed in all three patients. Target enrichment was performed with 0.5 μg of genomic DNA using the Sure SelectXT Human All Exon kit version 6 (Agilent Technologies, Santa Clara, CA, USA) and sequenced on the HiSeq2500 platform (Illumina, San Diego, CA, USA) as paired-end 125-bp reads with 60× coverage. Base calling was performed with the Illumina pipeline, with default parameters. Sequence reads were aligned to the reference genome (hg19) using Burrows-Wheeler Aligner (BWA). Variant calling was performed with DNAnexus software (DNAnexus Inc., Mountain View, CA; www.dnanexus.com). Filtering of called variants was performed in several steps using Ingenuity Variant Analysis (www.ingenuity.com/products/variant-analysis). We analysed 65 genes associated with cardiomyopathy (genes available on request). Further filtering was done to predict pathogenic variants using SIFT and PolyPhen2, the level of conservation and how common the variants are in the population (using 1000 Genome (http://www.1000genomes.org/); NHLBI Exome Sequencing Project (ESP) (http://evs.gs.washington.edu/EVS/); The Exome Aggregation Consortium (ExAC, http://exac.broadinstitute.org/)) to reduce the number of variants.

### Protein expression—western blot

Western blot was performed on protein extract from cryostat sections of a cardiac muscle biopsy specimen from patient 1, with or without alpha-amylase treatment. The alpha-amylase treatment was performed by incubating the cryostat section with 10 μg/ml human alpha-amylase (Sigma-Aldrich, St Louis, MO) in a total volume of 10 μl phosphate-buffered saline, pH 6.5 for 1 h at 37 °C. The protein extractions were performed by denaturing the samples using Laemmli sample buffer with 5 % β-mercaptoethanol, incubating 4 min at 95 °C and a final centrifugation for 10 min. The supernatants including protein were loaded and separated on 10 % Bis-Tris gel (Novex; Life Technologies, Grand Island, NY) followed by electroblotting. The membranes were incubated with primary antihuman glycogenin-1 N-terminal antibody M07 clone 3B5 (Abnova, Taipei, Taiwan; 1:500). Western Breeze Chromogenic kit (Life Technologies) was used for antibody detection.

### Cell-free glycogenin-1 expression and in vitro glucosylation

A cell-free protein expression system was used to analyse the autoglucosylation ability of the mutant protein. Site-directed mutagenesis of the glycogenin-1 sequence (NM_001184720) inserted in a pEXP5-NT/TOPO vector (Life Technologies, Carlsbad, CA) was performed using the QuikChange II site-directed mutagenesis protocol (Agilent Technologies, Santa Clara, CA, USA). Sanger sequencing confirmed the correct sequence. The FastLane *Escherichia coli* protein synthesis kit (RiNA, Berlin Germany) was used to express the mutated and wild type glycogenin-1 according to the manufacturer’s protocol. The expressed glycogenin-1 proteins were allowed to autoglucosylate in N-2-hydroxyethylpiperazine-N-2-ethanesulfonic acid (50 mM, pH7.5), MnCl_2_ (5 mM), dithiothreitol (0.5 mM) and 0.1 M uridine diphosphate (UDP)-glucose (Calbiochem, San Diego, CA) for 1 h at 30 °C (Hurley et al 2005). Half of the protein extract was treated with 10 μg/ml human alpha-amylase (Sigma-Aldrich, St Louis, MO) for 1 h at 37 °C. Western blot was performed on the protein extract with or without alpha-amylase treatment, denatured using Laemmli sample buffer with 5 % β-mercaptoethanol and separated on a 10 % Bis-Tris gel (Novex, Life Technologies, Grand Island, NY) followed by electroblotting. The membrane was incubated with primary antihuman glycogenin-1 N-terminal antibody M07 clone 3B5 (Abnova, Taipei, Taiwan; 1:500). Western Breeze Chromogenic kit (Life Technologies) was used for antibody detection.

## Results

### Morphological investigations

Endomyocardial biopsy of patient 1 showed storage of periodic acid-Schiff (PAS) positive material that was partially resistant to digestion by alpha-amylase in most cardiomyocytes (Fig. 2a-c). By electron microscopy this material appeared as normal glycogen but in many regions it was composed of filamentous or amorphous material (Fig. 2d-e). Skeletal muscle biopsy demonstrated variability of muscle fibre size and scattered muscle fibres with accumulation of PAS positive material. These fibres were frequently but not consistently devoid of normal glycogen (Fig. 2f).

**Fig. 2:**
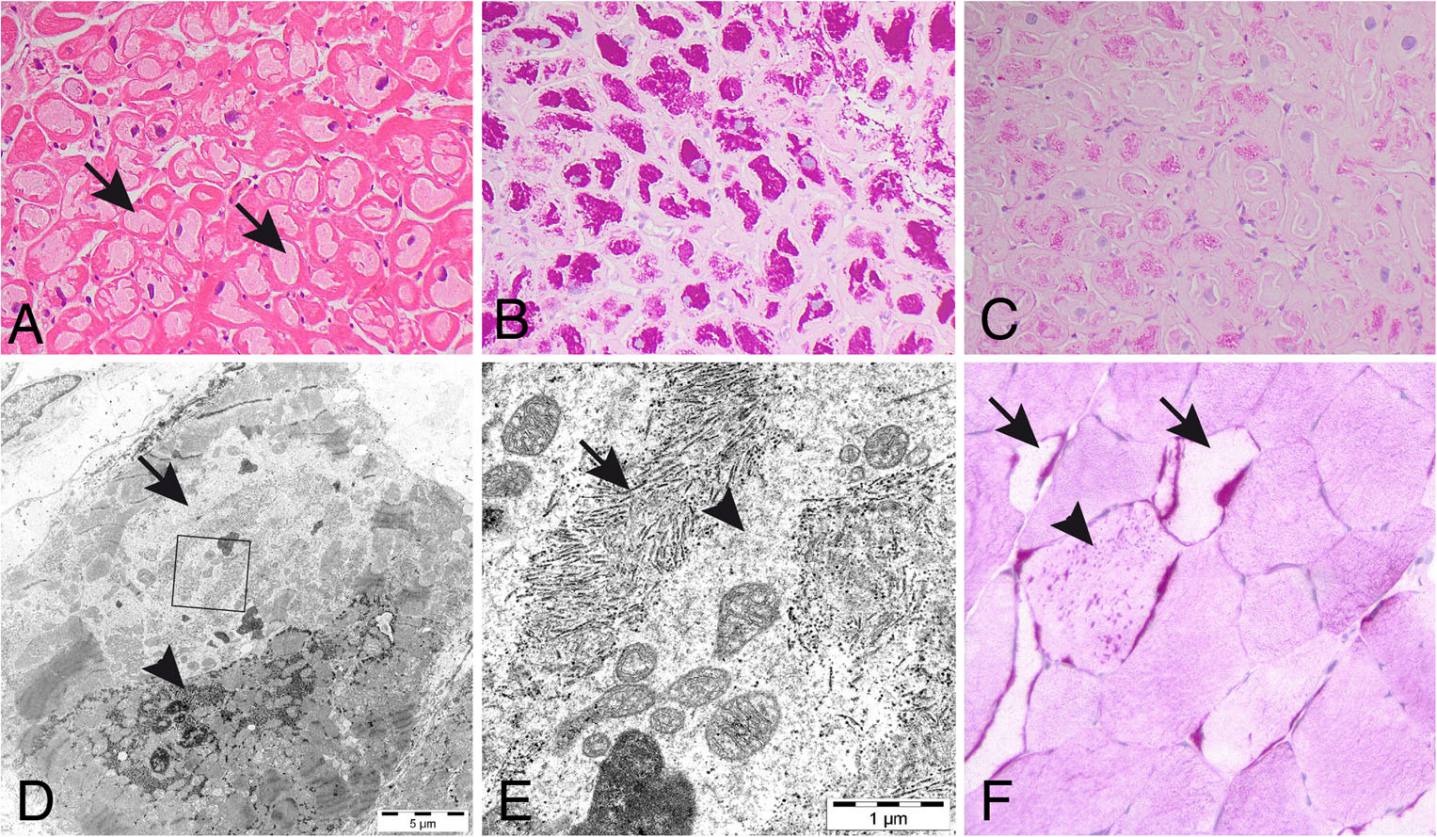
Endomyocardial and skeletal muscle biopsy of patient 1 with abnormal glycogen storage; (**a**) in virtually every cardiomyocyte there is a large central region with storage material (arrows; hematoxylin and eosin); (**b**) the storage material is intensely positive in PAS staining; (**c**) it is to a minor degree resistant to alpha-amylase treatment; (**d**) electron microscopy demonstrating accumulation of glycogen with normal appearance (arrow-head) as well as paler regions with storage of other type of material (arrow) that at higher magnification (e, inset in Fig. 1d) has a fibrillar (arrows) or unstructured (arrow-head) appearance. There is also some normal glycogen particles in addition to mitochondria and lipofuscin in this image; (**f**) skeletal muscle biopsy demonstrating by PAS staining that some fibres that look pale show accumulation of PAS positive material (arrows). There are also PAS positive inclusions in fibres with normal glycogen content (arrow-head)

In patient 2 an endomyocardial biopsy showed abnormal hypertrophied myocytes with extensive vacuolation replacing most of the cell (Suppl. Fig. 1). There was no evidence of inflammation, no vasculitis and no abnormal infiltrates. PAS-staining was positive with partial removal by diastase. Electron microscopy was not done.

Similarly, an endomyocardial biopsy from patient 3 showed myocyte enlargement with irregular hyperchromatic nuclei (Suppl. Fig. 2a, b). There was marked cytoplasmic vacuolation. PAS-staining was positive. Mitochondria were of normal size and morphology and were displaced by glycogen. Electron microscopy confirmed the storage of non-lysosomal glycogen, with displacement of nuclei and mitochondria (Suppl. Fig. 2c).

### Molecular genetic analysis

All three patients (Fig. 3a) were homozygous for a c.304G > C, p.Asp102His mutation in exon 3 of the *GYG1* gene (NM_004130.3) (Fig. 3b, c). The substitution of a negatively charged aspartic acid with a positively charged histidine was predicted to be highly pathogenic both by PolyPhen-2 with a score of 1.00 and SIFT function prediction.

**Fig. 2:**
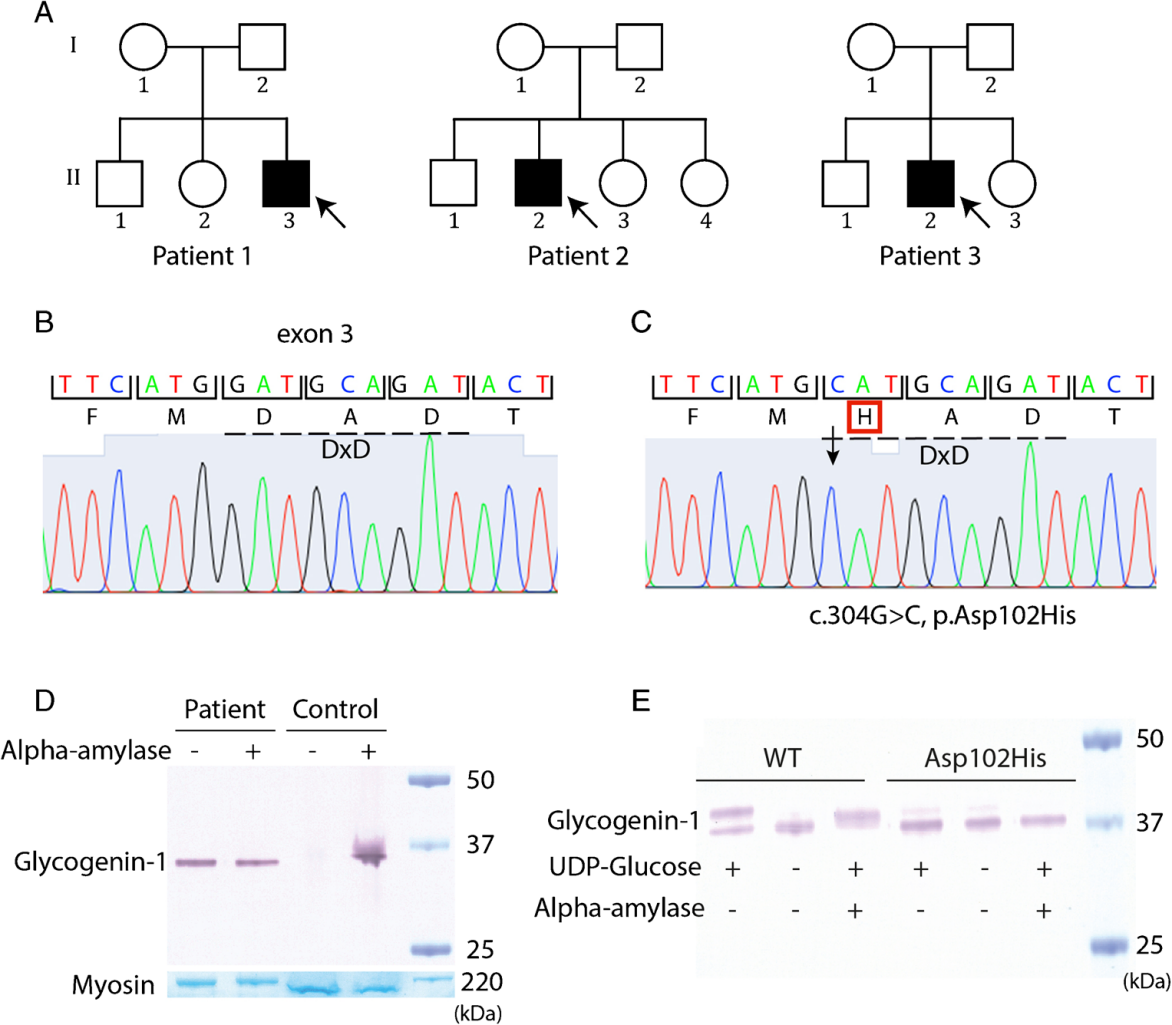
Genetic and protein analysis. **a** Pedigrees of the families. **b** Chromatogram of normal *GYG1* DNA sequence with the DxD motif indicated by hatched line. **c** Chromatogram demonstrating a homozygous missense mutation in *GYG1* (c.304G > C, p.Asp102His), the hatched line shows that the DxD motif is mutated. **d** Western blot analysis of glycogenin-1 on protein extracted from cardiac muscle biopsy specimen from patient 1 and a control sample performed without (−) or with alpha-amylase (+) treatment. In patient 1 glycogenin-1 was detected both without and with alpha-amylase treatment compared to control sample where glycogenin-1 was only detected after alpha-amylase treatment. The band corresponding to myosin heavy chain was used as loading control. **e** Cell-free autoglycosylation in vitro revealed that the protein with the mutation p.Asp102His was not able to autoglycosylate demonstrated with only one band after addition of uridine diphoshate (UDP) glucose (+) compared to wild type (WT) protein which was able to autoglycosylate demonstrated with two bands corresponding to both unglucosylated (lower band) and glycosylated (upper band) glycogenin-1. Unglucosylated glycogenin-1 weighs approximately 1 kDa less than free autoglucosylated protein. After alpha-amylase treatment the short glucose polymers are cleaved off resulting in only one band for WT compared to p.Asp102His which was not affected because of absence formation of short glucose polymers

The affected amino acid is in a highly conserved position among species with a conservation phyloP p-value of 1.000E-6. This variant is detected in 0.13 % in NHLBI ESP and with a 1000 Genomes frequency of 0.04 %.

Sixty-five candidate genes associated with cardiomyopathy were screened in all three patients. Most called variants after filtering were predicted to be likely benign or with uncertain significance according to computed ACMG Guidelines classification. Only a *MYH6* variant of uncertain importance was detected in patient 3. This variant was also detected in his asymptomatic mother. The only clearly pathogenic variant was the homozygous *GYG1* c.304G > C mutation in all three patients.

Genetic testing of family members revealed that the sister of patient 1 was also homozygous for the *GYG1* c.304G > C mutation. She was 53 years of age and had no clinical symptoms. Her ECG and echocardiogram were within normal limits, and MRI was essentially normal with no evidence of any abnormal signal on the delayed enhancement images. Neither of the two sisters of patient 2 had known cardiac disease. One was heterozygous for the c.304G > C allele and the other had normal *GYG1* sequence. The mother of patient 3 was heterozygous for the c.304G > C allele. No other family members agreed to genetic testing.

### Protein expression—western blot

In patient 1 the expression of glycogenin-1 in cardiac muscle tissue was studied by western blot analysis either without or with alpha-amylase treatment, to cleave off the glucose in the glycogen particles (Fig. 3d). In normal individuals, there is no, or very little, free glycogenin-1, since it is part of glycogen particles and therefore it can only be detected by western blot analysis after alpha-amylase treatment. In contrast, glycogenin-1 was detected both with and without alpha-amylase treatment in the patient demonstrating that it was not glucosylated.

### Cell-free expression and in vitro glucosylation

A normal gel shift, of approximately 1 kD after addition of UDP-glucose, was demonstrated for wild type glycogenin-1, indicating that it was autoglucosylated (Fig. 3e). Alpha-amylase treatment of the recombinant autoglucosylated wild type glycogenin-1 resulted in a reduction of the molecular weight, further confirming that the observed gel shift was due to autoglucosylation by the addition of 8–14 glucose residues. The in vitro autoglucosylation assay for the glycogenin-1 variant p.Asp102His did not demonstrate this expected gel shift, indicating that it had lost the ability to autoglucosylate.

## Discussion

Muscle GSD XV that is caused by variants in *GYG1* belongs to a group of disorders of glycogen metabolism with storage of polyglucosan in muscle and other tissues (Hedberg-Oldfors and Oldfors 2015). Several human genes are known to be associated with polyglucosan storage in muscle including *GYG1*, *GBE1*, *RBCK1* (*HOIL-1*), *PFKM*, *EPM2A*, *EPM2B* (*NHLRC1*), *PRDM8* and *PRKAG2*. Two of these genes, *GYG1* and *GBE1* encode for enzymes involved in glycogen synthesis. Neuromuscular forms of branching enzyme (*GBE1*) deficiency presents in many different clinical variants the most severe being fetal akinesia deformation sequence (FADS) but may also present with milder phenotypes with onset in childhood or adulthood (Oldfors and DiMauro 2013). The classic presentation is characterized by progressive liver cirrhosis leading to death in childhood unless liver transplantation is performed. So-called adult polyglucosan body disease manifests as a neurologic disorder with onset at 40–60 years of age with neurogenic bladder, peripheral neuropathy and central nervous system involvement causing spastic gate and frequently cognitive impairment (Mochel et al 2012). In branching enzyme deficiency there is polyglucosan accumulated in the affected organs and tissues. RBCK1 deficiency is a rare disease that is characterized by onset usually in childhood or adolescence of muscle weakness and rapidly progressive cardiomyopathy (Nilsson et al 2013). Some patients may also have immunodeficiency and autoinflammation (Boisson et al 2012). Polyglucosan is accumulated in several tissues including skeletal and cardiac muscle. Lafora disease is a lethal neurodegenerative disorder affecting adolescents (Minassian 2001). It is characterized by intractable myoclonic seizures, ataxia, visual hallucinations, cognitive decline and later dementia. Lafora bodies that are composed of polyglucosan are found in nerve cells and also in muscle. Two genes are associated with Lafora disease; *EPM2A* (laforin) and *EPM2B* (malin) and a third gene *PRDM8* that is associated with an early onset variant of Lafora body disease has been described (Turnbull et al 2012). In addition to these disorders muscle phosphofructokinase (*PFKM*) and AMP-activated protein kinase (*PRKAG2*) deficiency are associated with occasional polyglucosan inclusions in skeletal and cardiac muscle respectively (Arad et al 2005; Malfatti et al 2012).

The pathogenesis of polyglucosan accumulation in muscle is not completely known but is probably different in the various polyglucosan body diseases. In Lafora disease there is evidence that lack of laforin, which functions to remove phosphate from glycogen, or deficient removal of laforin from glycogen by malin makes the glycogen prone to aggregation as polyglucosan (Tiberia et al 2012). An imbalance of the activities of branching enzyme and glycogen synthase has been proposed as important for polyglucosan formation in equine polysaccharide storage myopathy (PSSM1) that is due to hyper-activity of glycogen synthase (*GYS1*) secondary to a dominant missense variant (McCue et al 2008).

We describe three unrelated patients homozygous for a missense mutation in the *GYG1* gene, presenting with glycogen storage cardiomyopathy and heart failure in two of them. There are several lines of evidence that the homozygous c.304G > C mutation causes the disease. First, three unrelated patients with the same homozygous mutation displayed a similar cardiac phenotype. Second, the mutation alters a highly conserved amino acid located in a motif essential for function. Third, the mutation abolishes the normal autoglucosylation. Fourth, a previously reported patient with other pathogenic *GYG1* mutations had similar abnormal glycogen storage in the heart. Fifth, several other *GYG1* mutations have been shown to cause similar abnormal glycogen storage in skeletal muscle. Sixth, whole-exome sequencing did not reveal any pathogenic mutations in other genes associated with cardiomyopathy.

This cardiomyopathy associated with a glycogenin-1 mutation but without skeletal muscle weakness differs from the previously described cases who typically had a rather late onset skeletal myopathy without cardiomyopathy (Malfatti et al 2014; Colombo et al 2016; Fanin et al 2015; Luo et al 2015; Akman et al 2016). Altogether 22 patients have been diagnosed with *GYG1* associated skeletal- or cardiomyopathy (for details of 19 previously reported patients see Suppl. Table 1). The majority of patients had adult onset (40–65 years), slowly progressive myopathy with muscle weakness that was predominantly proximal and symmetrical, causing walking difficulties with myopathic gait, difficulty in climbing stairs and hyperlordosis. Some patients have become wheelchair-dependent. In two patients there was a suspicion of childhood onset. Exercise intolerance, myalgia and distal muscle weakness were reported in a few patients. EMG was myopathic with a neurogenic component in a few cases. Serum-CK was mildly elevated in only five patients. In the majority of patients, cardiac examination including echocardiography and ECG was normal. Muscle pathology showed characteristic alterations, similar to patient 1 described in this paper, with PAS positive inclusions in a proportion of the muscle fibres that were depleted of normally distributed glycogen. These PAS positive inclusions were partly resistant to digestion by alpha-amylase. In addition, muscle tissue was atrophic with muscle fibre size variability and increase in interstitial fat and fibrous connective tissue. One patient had depletion of glycogen alone, without inclusions. This patient also had cardiomyopathy. To date, no impact on liver function has been reported, and glycogen storage such as seen in the biopsy from patient 1 may be subclinical. This may be due to glycogenin-2 that is expressed in the liver, although the importance of glycogenin-2 has been debated (Irgens et al 2015). In previously described patients, at least 32 loss-of-function (nonsense and splice site) variants in the 38 *GYG1* alleles were identified. The most common variant is c.143 + 3G > C that has been identified in 25 alleles.

The clinical variability of *GYG1* mutations therefore seems to include pure skeletal myopathy and pure cardiomyopathy that may lead to cardiac failure. In addition, the first described case with a glycogenin-1 associated glycogen storage disease presented with cardiac arrest caused by arrhythmia (Moslemi et al 2010). This patient had the same type of storage of abnormal glycogen in cardiomyocytes as the cases presented in the present report. However, he also had muscle weakness and depletion of glycogen in skeletal muscle, which further broadens the clinical spectrum. It is noteworthy that the sister of patient 1, who is also homozygous for the *GYG1* c.304G > C mutation does not show signs or symptoms of cardiac or skeletal muscle disease. This is in line with the previously noted great variability of clinical expression of even the same *GYG1* mutation. The homozygous *GYG1* c.143 + 3G > C mutation showed variability of age at onset from childhood to age 60 years in 11 reported patients (Malfatti et al 2014; Colombo et al 2016; Fanin et al 2015; Akman et al 2016).

Patients with glycogenin-1 deficiency and cardiomyopathy demonstrated extensive late gadolinium enhancement by cardiac MRI. This finding in addition to endomyocardial biopsy demonstrating glycogen storage may be a clue to the correct diagnosis, which can be verified by glycogenin-1 gene analysis. In the future, the addition of *GYG1* to cardiomyopathy gene panels will simplify the identification of these patients. Considering the relative high frequency of the *GYG1* c.304G > C allele it may be anticipated that many more glycogenin-1 associated cardiomyopathy patients will be identified. The mutation was identified in 16/8584 alleles in European Americans in the NHLBI ESP (Exome Variant Server, NHLBI GO Exome Sequencing Project (ESP), Seattle, WA; URL: http://evs.gs.washington.edu/EVS/) (June, 2015) and in 95/66728 alleles in European Non Finnish population in the ExAC database. All three patients were homozygous for the c.304G > C, p.Asp102His mutation in exon 3 of the *GYG1* gene. All patients are of British descent so it may be speculated that there is a founder mutation. Further analysis on the segregation of haplotypes may clarify this issue.

**Fig. 4:**
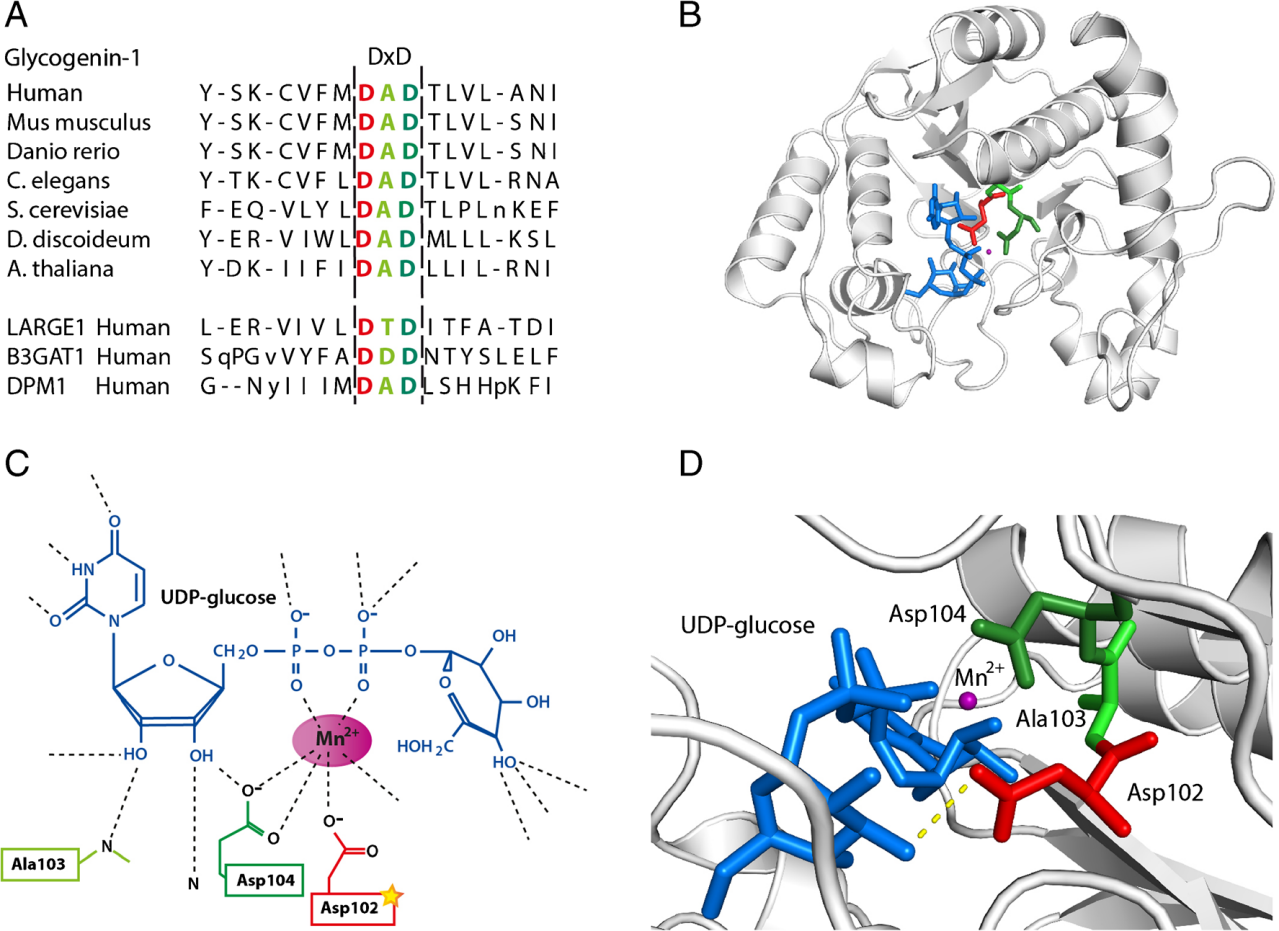
The glycogenin-1 protein. **a** Alignment of glycogenin-1 proteins in different species. The bottom three lines demonstrating proteins belonging to other gene families with DxD motif. The amino acid affected by the mutation in red and within the hatched line is the conserved DxD motif. **b** Illustration of the stereo view of glycogenin-1 with the amino acids in the DxD motif marked with colours, the affected aspartic acid at position 102 in red, alanine and aspartic acid at positions 103 and 104 respectively in green. UDP-glucose is labelled in blue and Mn^2+^ is labelled in purple. This figure was generated using The PyMOL Molecular Graphics System, Version 1.7.2.1 Schrödinger, LLC. **c** Schematic illustration of the interaction between glycogenin-1, UDP-glucose and Mn^2+^. Amino acids within the DxD motif are included and labelled in colours, the amino acid affected by mutation is in red (modified from Gibbons et al 2002). **d** Higher magnification of the stereo view demonstrating the UDP-glucose, Mn^2+^ and the DxD motif. The polar contact between UDP-glucose and Asp102 is demonstrated with the yellow-hatched line. The same colour scheme is used throughout the figure

The mutated glycogenin-1 protein was expressed in cardiac muscle. The affected amino acid is in a highly conserved position among species (Fig. 4a-b). It is located in the DxD motif, which is essential for the coordination of the catalytic divalent cation Mn2+ (Fig. 4c) (Gibbons et al 2002). Glycogenin-1 folding creates a cleft for the sugar-donor UDP-glucose and Mn^2+^ and the DxD is located in the putative active site of the enzyme (Persson et al 2001; Gibbons et al 2002).

Glycogenin-1 belongs to glycosyltransferase family 8, which includes galactinol synthases, galactose transferases and lipopolysaccharide glucose transferases (Campbell et al 1997). The DxD motif is well conserved and found in all family 8 glycosyltransferases (Persson et al 2001). Mutations of one of the Asp residues within the DxD motif are described to result in inhibition of glycosyl transferase activity in several different species (Busch et al 1998; Shibayama et al 1998). Other glycosyltransferases with DxD motifs that are involved in muscle diseases include acetylglucosaminyltransferase-like protein (*LARGE*) (Brockington et al 2005), a member of the N-acetylglucosaminyltransferase gene family, *B3GAT1* a Beta-1,3-glucuronyltransferase (Mitsumoto et al 2000) and *DPM1*, a member of the glycosyltransferase family 2 (Tomita et al 1998) (Fig. 4a).

In line with the predicted deleterious effect of the mutation, our results from western blot analysis of protein extract from cardiac tissue of patient 1 and the in vitro autoglucosylation assay demonstrated loss of autoglucosylation caused by the p.Asp102His *GYG1* mutation. In cardiac tissue glycogenin-1 was detected both without and with alpha-amylase treatment in protein extract from patient 1 compared to control sample where glycogenin-1 was only detected after alpha-amylase treatment, thus demonstrating that it did not initiate glycogen synthesis in the patient. This was confirmed by the in vitro autoglycosylation assay, which demonstrated that the autoglycosylation activity was abolished by the replacement of Asp at position 102 with His.

The expression of a non-functional glycogenin-1 was also described in a previous case with cardiomyopathy (Moslemi et al 2010). Analysis of glycogenin-1 expression has been performed in only a few cases with isolated skeletal muscle weakness. In a series of five patients with skeletal myopathy reported by Malfatti et al (2014) there was either a markedly reduced amount or complete absence of glycogenin-1 protein in skeletal muscle. This may indicate that high expression of a non-functional mutated protein might be more deleterious to the heart than absence of the glycogenin-1 protein. One previously reported patient with clinically pure skeletal myopathy was compound heterozygous for the p.Asp102His *GYG1* mutation and a nonsense variant but no western blot was performed so it is not known to what extent the mutant protein was expressed in that case (Malfatti et al 2014). Although our patients with cardiomyopathy had no muscle weakness, a biopsy of patient 1 demonstrated involvement of skeletal muscle. Thus, there may be a combined sub-clinical involvement only disclosed by a biopsy, which may be of diagnostic value in some cases.

The accumulation of apparently normal as well as abnormal glycogen forming amorphous or fibrillar material in the myocardium is intriguing in view of the abolished glycogenin-1 activity. The accumulated glycogen is partly alpha-amylase resistant and has a partly filamentous structure compatible with amylopectin-like material or polyglucosan. This type of storage in muscle is not unique for glycogenin-1 deficiency but is also found in deficiency of branching enzyme, phosphofructokinase and RBCK1 and in Lafora disease among others (Hedberg-Oldfors and Oldfors 2015). Therefore, multiple different gene mutations result in this type of abnormal storage. Future studies on the detailed composition of the storage material in these diseases may unravel the pathogenesis.

The cause of cardiomyopathy and cardiac failure in glycogenin-1 deficiency is not fully understood but cellular overload of storage material leading to cell death and secondary fibrosis as demonstrated by MRI and histological investigations is a plausible explanation. A cytotoxic effect of storage material in addition to depletion of normal glycogen could be of importance for the pathophysiology both in skeletal and cardiac muscle. Unlike some other metabolic disorders, e.g. Pompe disease (GSD II) enzyme replacement therapy is at present not available in glycogenin-1 deficiency. Although there are no reports of dietary management for glycogenin-1 defects, triheptanoin (a seven-carbon triglyceride) is currently in clinical trials for other glycogen storage (GDS V, NCT02432768) and polyglucosan body (GSD IV, NCT00947960) disorders and so may have a potential future role in management. Further research on the pathogenic mechanism may identify goals for specific therapeutic interventions.

In conclusion we have identified three unrelated patients homozygous for a missense *GYG1* mutation presenting with cardiomyopathy and progressive dilatation and heart failure in two of them, demonstrating that glycogenin-1 deficiency should be considered a cause of cardiomyopathy in adults.

## Electronic supplementary material

Below is the link to the electronic supplementary material.

ESM 1 (DOCX 1277 kb)

ESM 2 (DOCX 2826 kb)

ESM 3 (DOCX 136 kb)
