# Supplementary material for: Cardiomyopathy as presenting sign of glycogenin-1 deficiency—report of three cases and review of the literature
Source: J Inherit Metab Dis. 2016 Oct 7;40(1):139–49. doi: 10.1007/s10545-016-9978-1 (PMC5203857; doi:10.1007/s10545-016-9978-1)
Supplement: Supplementary file 1 — (DOCX 1277 kb) [file 10545_2016_9978_MOESM1_ESM.docx]

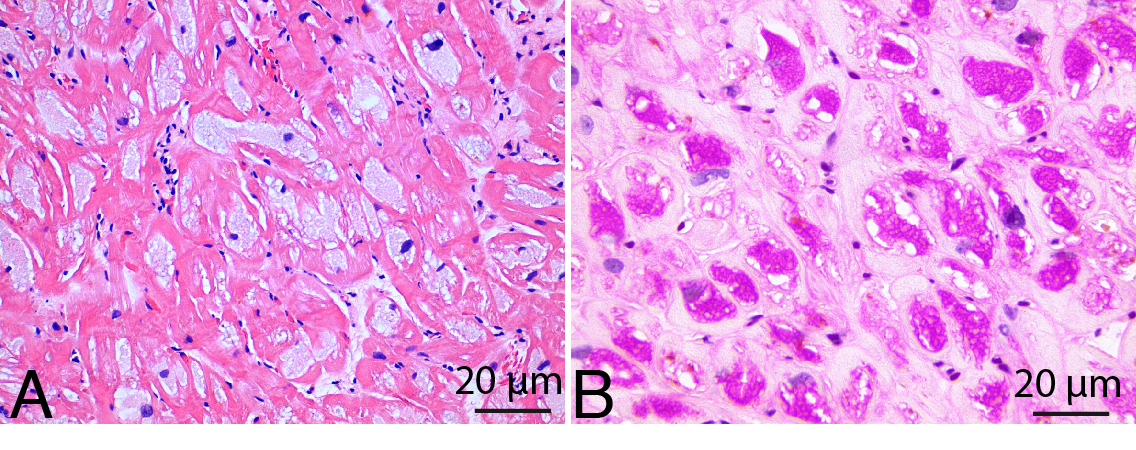


**Suppl. Figure 1.** Endomyocardial biopsy of patient 2 demonstrating vaculation of the cardiomyocytes. (A) Hematoxylin and eosin staining. (B) The vacuoles show storage of PAS positive material.
