# Supplementary material for: Cardiomyopathy as presenting sign of glycogenin-1 deficiency—report of three cases and review of the literature
Source: J Inherit Metab Dis. 2016 Oct 7;40(1):139–49. doi: 10.1007/s10545-016-9978-1 (PMC5203857; doi:10.1007/s10545-016-9978-1)
Supplement: Supplementary file 2 — (DOCX 2826 kb) [file 10545_2016_9978_MOESM2_ESM.docx]

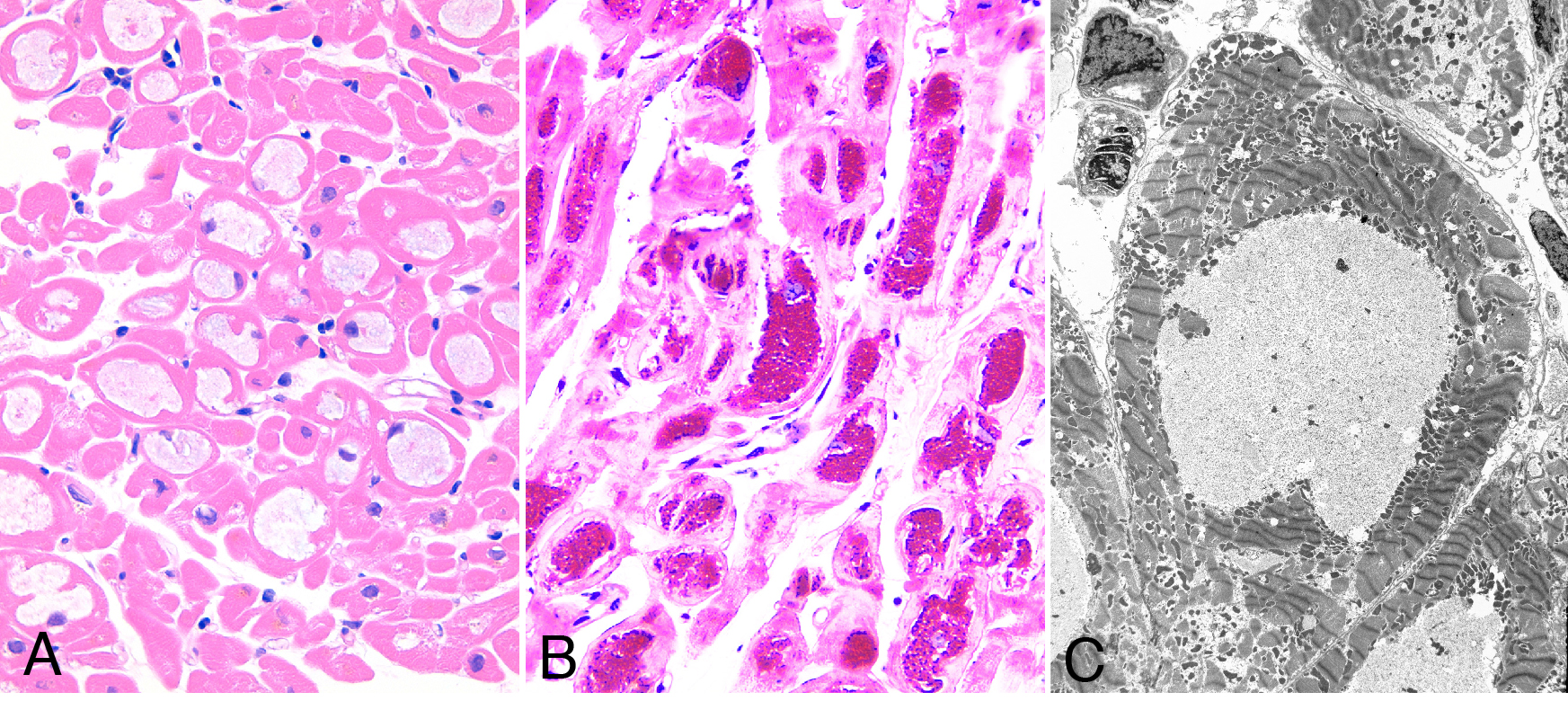


**Suppl. Figure 2.** Endomyocardial biopsy of patient 3 showing vaculation of the cardiomyocytes. (A) Hematoxylin and eosin staining. (B) The vacuoles show storage of PAS positive material. (C) Electron microscopy demonstrating accumulation of glycogen in a vacuole in the center of a cardiomyocyte.
