# Supplementary material for: Cardiomyopathy as presenting sign of glycogenin-1 deficiency—report of three cases and review of the literature
Source: J Inherit Metab Dis. 2016 Oct 7;40(1):139–49. doi: 10.1007/s10545-016-9978-1 (PMC5203857; doi:10.1007/s10545-016-9978-1)
Supplement: Supplementary file 3 — (DOCX 136 kb) [file 10545_2016_9978_MOESM3_ESM.docx]

Supplementary Table 1. Summary of reported cases with glycogenin-1 deficiency

|  | This study | | | Moslemi et al, 2010 | Malfatti et al, 2014 | | | | | | |
| --- | --- | --- | --- | --- | --- | --- | --- | --- | --- | --- | --- |
| Gender | M | M | M | M | M | F | M | F | M | F | F |
| Patient origin | New Zealand/British | British | British | Swedish | French | US | US | Spanish | German | German | Belgian |
| Age at onset | 34 | 46 / 50 | 23 | Probably childhood | 17 | Probably childhood | 39 | 65 | 62 | 61 | 49 |
| Initial symptoms | Shortness of breath,  chest pain on exertion,  lethargy and palpitations | CVA / Chest pain,  sweatiness and  palpitations | Shortness of breath and  chest pain | Shortness of breath and upper arm weakness | Difficulty running | Trouble rising from squatting | Difficulty walking | Difficulty climbing stairs and walking | Difficulty climbing stairs and walking | Loss of grip strength in both hands | Difficulty climbing stairs and loss of grip strength in right hand |
| Age at exam. yr |  |  |  | 27 | 26 | 50 | 43 | 72 | 72 | 66 | 57 |
| Clinical features of muscle weakness | Not present | Not present | Not present | Cardiac arrhythmias, weakness in upper arms, neck flexion and foot dorsiflexion | Hip girdle muscle weakness, atrophy and fatigability | Shoulder, hip girdle and distal anterior leg muscle weakness | Hip girdle, tibial and peroneal muscle weakness | Shoulder and hip girdle muscle weakness | Hip girdle and thigh muscle weakness and atrophy; milder asymmetric weakness of shoulder girdle and upper arm muscles | Intrinsic hand muscle weakness and atrophy; mild extrinsic finger extensor muscle weakness | Shoulder and hip girdle muscle weakness; wrist and finger extensors, interosseus, foot and toe extensor paralysis |
| Weakness distribution | No weakness | No weakness | No weakness | Proximal/distal | Proximal | Proximal/distal | Proximal/distal | Proximal/distal | Proximal | Distal | Proximal/distal |
| Clinical course | Progressive | Progressive | Progressive | Progressive | Progressive | Progressive | Progressive | Progressive | Progressive | Progressive | Progressive |
| Serum CK | Elevated | Normal | Normal | Normal | Elevated | Normal | Normal | Normal | Normal | Normal | Normal |
| Cardio-myopathy | Yes | Yes | Yes | Yes | No | No | No | No | No | No | No |
| EMG | nd | nd | nd | nd | Myopathic | Myopathic | Myopathic | Myopathic | Myopathic and neurogenic | Myopathic | Myopathic |
| Polyglucosan in  muscle biopsy | Yes | nd | nd | No  (deficit of glycogene) | Yes | Yes | Yes | Yes | Yes | Yes | Yes |
| Polyglucosan bodies on EM in  muscle biopsy | nd | nd | nd | No  (glycogene depletion) | Yes | Yes | Yes | Yes | Yes | Yes | Yes |
| DNA analysis | Homozygous c.304G>C | Homozygous c.304G>C | Homozygous c.304G>C | Compound heterozygous  c.248C>T  c.484delG | Homozygous c.143+3G>C | Homozygous c.143+3G>C | Compound heterozygous c.304G>C, c.749G>A | Homozygous c.46G>C | Compound heterozygous c.143+3G>C, c.7G>C | Homozygous c.484delG | Compound heterozygous c.143+3G>C c.970C>T |
| RNA analysis | nd | nd | nd | nd | Yes | Yes | nd | Yes | Yes | Yes | Yes |
| Predicted protein change | p.Asp102His | p.Asp102His | p.Asp102His | p.ThrMet83  p.Thr163Aspfs*5 | p.Asp3Glufs*4 | p.Asp3Glufs*4 | p.Asp102His  p.Trp250* | p.Ala16Pro | p.Asp3Glufs*4  p.spl | p.Thr163Aspfs*5 | p.Asp3Glufs*4 p.Arg324* |

Supplementary Table 1. continued

| Reference | Luo et al, 2015 | Akman et al, 2015 | | | | | Fanin et al, 2015 | | | Colombo et al, 2015 | |
| --- | --- | --- | --- | --- | --- | --- | --- | --- | --- | --- | --- |
| Gender | F | M | M | F | F | F | M | F | F | F | F |
| Patient origin | Chinese | Italian | Italian | Italian | Italian | Italian | Italian | Italian | Italian | Italian | Italian |
| Age at onset, yr | 39 | 40 | 53 | 55 | 60 | 45 | 66 | 52 | 50 | 30 | 53 |
| Initial symptoms | Difficulty combing her hair and climbing stairs | Exercise intolerance and myalgia | Difficulty climbing stairs and walking | Weakness in cervical and pelvic girdles | Weakness in scapular and pelvic girdles | Difficulty climbing stairs and walking; exercise intolerance | Difficulty climbing stairs and walking | Difficulty climbing stairs | Limb myalgia | Arm abduction weakness | Difficulty climbing stairs; arm abduction weakness |
| Age at exam. yr | 46 | 65 | 65 | 77 | 80 | 65 | 74 | 60 | 70 | 71 | 64 |
| Clinical features | Neck flexor and asymmetric proximal limb weakness; reduced grip strength; exercise intolerance | Weakness in scapular and pelvic girdles; waddling gait; hyperlordosis | Waddling gait; right winging scapula; Gowers sign; hyperlordosis | Waddling gait; hyperlordosis; kyphosis; severe proximal muscle weakness | Waddling gait; hyperlordosis; severe proximal muscle weakness | Waddling gait; hyperlordosis; severe lower limb weakness | Waddling gait; weakness in shoulder and pelvic girdles; pes cavus | Waddling gait; weakness in proximal limb muscles; pes cavus | Waddling gait; Gowers sign; shoulder girdle weakness, quadriceps wasting | Severe proximal and distal muscle weakness; facial weakness; wheelchair bound; | Waddling gait; Gowers sign; scapular winging; weakness in shoulder and pelvic girdles |
| Weakness distribution | Proximal | Proximal | Proximal | Proximal | Proximal | Proximal | Proximal | Proximal/Distal | Proximal | Proximal/Distal | Proximal |
| Clinical course | Progressive | Progressive | Progressive | Progressive | Progressive | Progressive | Progressive | Progressive | Progressive | Progressive | Progressive |
| Serum CK | Normal | Normal | Elevated | Normal | Normal | Normal | Elevated | Elevated | Normal | Normal | Normal |
| Cardio-myopathy | No | No  (ischemic heart disease) | No  (dilation of aortic root; slight left atrial dilation) | No  (sclerosis of aortic valve; slight mitral and tricuspid insufficiency) | No  (slight mitral and tricuspid insufficiency; hypertensive cardiomyopathy) | No  (slight mitral and tricuspid insufficiency) | No  (ischemic cardiomyopathy) | No | No | No | No |
| EMG | Myopathic | Myopathic | Neurogenic | Myopathic | Myopathic | Myopathic | nd | nd | nd | Myopathic | Myopathic |
| PAS-positive inclusions | Yes | Yes | Yes | Yes | Yes | No | Yes | Yes | Yes | Yes | Yes |
| Polyglucosan bodies on EM | nd | Yes | Yes | Yes | Yes | Not found | Yes | Yes | Yes | Yes | Yes |
| DNA analysis | Homozygous  c.634C>T | Homozygous c.143+3G>C | Homozygous c.143+3G>C | Homozygous c.143+3G>C | Homozygous c.143+3G>C | Homozygous c.143+3G>C | Homozygous c.143+3G>C | Homozygous c.143+3G>C | Compound heterozygous c.143+3G>C, g.148717967C>G | Homozygous c.143+3G>C | Homozygous c.143+3G>C |
| RNA analysis | nd | nd | nd | nd | nd | nd | nd | nd | Yes | nd | nd |
| Predicted protein change | p.His212Tyr | p.Asp3Glufs*4 | p.Asp3Glufs*4 | p.Asp3Glufs*4 | p.Asp3Glufs*4 | p.Asp3Glufs*4 | p.Asp3Glufs*4 | p.Asp3Glufs*4 | p.Asp3Glufs*4  p.spl | p.Asp3Glufs*4 | p.Asp3Glufs*4 |

nd: Not described

EM: electron microscopy
